# Human NKG2D-ligands: cell biology strategies to ensure immune recognition

**DOI:** 10.3389/fimmu.2012.00299

**Published:** 2012-09-25

**Authors:** Lola Fernández-Messina, Hugh T. Reyburn, Mar Valés-Gómez

**Affiliations:** Departamento de Inmunología y Oncología, Centro Nacional de Biotecnología, Consejo Superior de Investigaciones CientíficasMadrid, Spain

**Keywords:** innate immunity, NKG2D receptor, MICA/B, ULBP, shedding, exosomes, immune evasion

## Abstract

Immune recognition mediated by the activating receptor NKG2D plays an important role for the elimination of stressed cells, including tumors and virus-infected cells. On the other hand, the ligands for NKG2D can also be shed into the sera of cancer patients where they weaken the immune response by downmodulating the receptor on effector cells, mainly NK and T cells. Although both families of NKG2D-ligands, major histocompatibility complex class I-related chain (MIC) A/B and UL16 binding proteins (ULBPs), are related to MHC molecules and their expression is increased after stress, many differences are observed in terms of their biochemical properties and cell trafficking. In this paper, we summarize the variety of NKG2D-ligands and propose that selection pressure has driven evolution of diversity in their trafficking and shedding, but not receptor binding affinity. However, it is also possible to identify functional properties common to individual ULBP molecules and MICA/B alleles, but not generally conserved within the MIC or ULBP families. These characteristics likely represent examples of convergent evolution for efficient immune recognition, but are also attractive targets for pathogen immune evasion strategies. Categorization of NKG2D-ligands according to their biological features, rather than their genetic family, may help to achieve a better understanding of NKG2D-ligand association with disease.

## IMMUNE ACTIVATION THROUGH NKG2D

The activation of the immune system mediated by engagement of NKG2D with its ligands is a crucial step in the regulation of both innate and adaptive immune responses. The promiscuous binding of a single receptor, NKG2D, to a set of related, but diverse proteins is remarkable and there has been much speculation on the evolution and functional significance of this diversity of NKG2D-ligands. The most widely accepted hypothesis to explain the existence of many NKG2D-ligands is that they have appeared during the evolution of the immune system in response to selection pressures exerted by pathogens or cancer (Eagle and Trowsdale, 2007). However, for this hypothesis to be valid there must be significant differences in the biochemistry and cell biology of the distinct NKG2D-ligands so that at least some ligands can still reach the cell surface despite the blockade of different cellular pathways in pathogen-infected or malignant cells. Initially, the different forms of membrane anchoring of the major histocompatibility complex class I-related chain (MIC) A/B and UL16 binding protein (ULBP) 1–3 molecules [the former, transmembrane (TM); the latter, glycosyl-phosphatidyl-inositol (GPI)-anchored] were thought to lead to differences in the cell biology of these proteins. However, identification of more ULBP genes and recent experiments studying the biochemistry and cell biology of various members of the two families of NKG2D-ligands have revealed common functional properties that are conserved between particular ULBP molecules and MIC alleles, but not within the different alleles at the MICA/B locus or the ULBP family. Those features shared between MICs and ULBPs may well represent examples of convergent evolution, presumably for reasons of maximal efficiency of immune recognition. On the other hand, these common features have the disadvantage of being attractive targets for pathogen immune evasion strategies. These observations suggest that the classification of NKG2D-ligands needs to be revisited and that consideration of similarities and differences between MIC and ULBP molecules may help to understand the biology of these proteins and therefore the impact of NKG2D-ligand expression on disease.

## THE NKG2D RECEPTOR

NKG2D is a type II TM protein that belongs to the C lectin-like family and is encoded on chromosome 12 in humans (Houchins et al., 1991), mapping within the NK gene complex and in the syntenic chromosome 6 in mice (Brown et al., 1997; for review, see Champsaur and Lanier, 2010). It is expressed as a homodimer on all NK cells and, in humans, it is also constitutively expressed on CD8^+^ αβ and γδ T cells from peripheral blood and intestinal intraepithelium (for review, see Raulet, 2003; Chan et al., 2006). Recently, it has also been described that expression of the NKG2D receptor can be induced on a small subset of CD4^+^ T cells (Groh et al., 2006; Saez-Borderias et al., 2006). NKG2D mediates both activating and co-stimulatory signals. In NK cells, NKG2D ligation is sufficient to trigger cell activation, whereas when expressed in CD8^+^ T cells, the interaction of the receptor with its ligands has a co-stimulatory function, similar to CD28. This effect is mediated through enhancement of cytokine production as well as signals that activate TCR driven cytotoxicity, but it is not sufficient to activate target cell lysis in the absence of TCR engagement (Bauer et al., 1999; Jamieson et al., 2002; Snyder et al., 2004), unless T cells have been previously activated by culture *in vitro* with IL-2 (Verneris et al., 2004). Consistent with these data, studies on intestinal intraepithelial lymphocytes incubated with IL-15, mimicking the conditions of coeliac disease, demonstrated that these lymphocytes are able to produce IL-10 and interferon (IFN)-γ after NKG2D ligation without TCR engagement (Meresse et al., 2004; Ebert, 2005). Indeed, NKG2D^+^ CD4^+^ T cells, not present in healthy individuals, have been reported to be involved in the patho-physiology of several immune-mediated diseases such as rheumatoid arthritis (Groh et al., 2003), Crohn’s disease (Allez et al., 2007), Wegener’s granulomatosis (Capraru et al., 2008), and human cytomegalovirus (HCMV) infection (Saez-Borderias et al., 2006).

The extracellular domain of the NKG2D receptor is involved in the interaction with its diverse ligands and its cytoplasmic tail lacks classic signaling sequences. Thus, an adaptor molecule is required to transduce the activation induced by ligand–receptor interaction. NKG2D has a charged amino-acid residue in its TM domain that mediates interaction with a complementary-charged amino-acid in the signaling polypeptide: DAP10 in humans (Wu et al., 1999) and either DAP10 or DAP12 in mice (Gilfillan et al., 2002). DAP10 is a TM signaling polypeptide that has an intracellular YxxM motif, which, upon tyrosine phosphorylation, couples the NKG2D/NKG2D-ligand complex to the phosphatidylinositol-3-kinase (PI3K)/Grb-2/Vav1 pathway (Upshaw et al., 2006), leading to activation. Noteworthy, the recruitment of both the effector molecule Vav-1 and the intermediate molecule Grb2 to DAP10 are necessary for cell-mediated cytotoxicity. Cell surface expression of the receptor can be modulated by cytokine secretion in the tissue microenvironment as well as by the presence of soluble NKG2D-ligands. For example, the γ-chain cytokines IL-2 and IL-15 rapidly increase the expression of both NKG2D and DAP10 on CD8^+^ T cells (Dhanji and Teh, 2003; Verneris et al., 2004; Dann et al., 2005; Maasho et al., 2005). Similarly, IL-15 in combination with TNF-α can induce the expression of NKG2D in the CD4^+^NKG2D^+^ T cells found in patients with rheumatoid arthritis mentioned previously (Groh et al., 2003). Moreover, IL-15 and IL-7 can maintain NKG2D surface expression after NKG2D co-stimulation of TCR activated CD8^+^ T cells (Maasho et al., 2005). On the other hand, exposure to other cytokines can produce a downmodulation of the NKG2D receptor. IL-21, produced by activated CD4^+^ T cells, which by its own activates both CD8^+^ T cells and NK cells promoting NKG2D-dependent killing of tumor cells (Takaki et al., 2005), when secreted in combination with IL-2 induces downregulation of NKG2D, thus silencing of the receptor-mediated immunosurveillance (Burgess et al., 2006). Similarly, TGF-β1, secreted by many types of cancer cells, reduces NKG2D surface expression, impairing tumor cytotoxic recognition by effector cells (Castriconi et al., 2003). Finally, cytokines such as IL-12 and IFN-β are associated with a reduction of NKG2D expression triggered by interaction with HCMV-infected dendritic cells (Muntasell et al., 2010).

## TWO FAMILIES OF NKG2D-LIGANDS BASED ON GENE LOCATION

**FIGURE 1:**
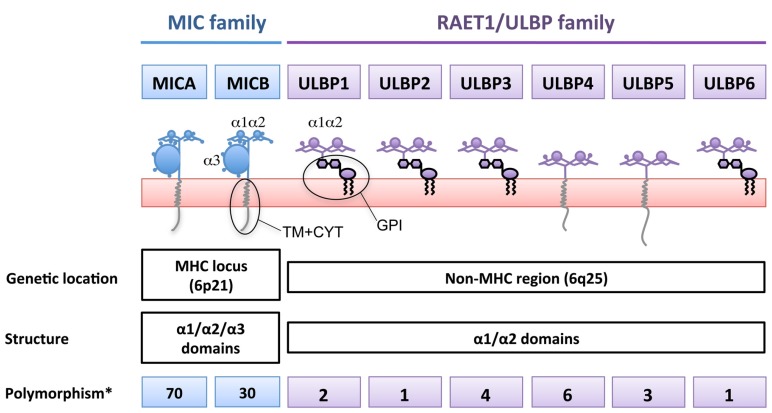
**The two genetic families of ligands for NKG2D.** NKG2D-ligands belong to two genetic families present in different arms of chromosome 6, giving rise to a large number of proteins with different biochemical properties. MICA/B are highly polymorphic genes while ULBPs only have a small number of variants due to single nucleotide polymorphisms. *Number of transcripts annotated in the Ensembl database (Release 66 – Feb 2012). For the ULBPs, family studies to show allelic segregation of these variants have not been performed.

Two families of ligands for the human NKG2D receptor have been described (**Figure 1**): the MICA/B encoded in the MHC region (Bahram et al., 1994; Bauer et al., 1999) and a second family of MHC class I-related proteins, the ULBPs, also known as retinoic acid early transcripts (RAETs), discovered while looking for ligands of the HCMV glycoprotein UL16 (Cosman et al., 2001), although only ULBP1, 2, and 6 bind UL16. ULBPs are also encoded on chromosome 6, but outside the MHC locus. Like conventional MHC class I molecules, MICA/B proteins contain α1, α2, and α3 domains, however, they do not associate with β2-microglobulin and they do not present peptides (Groh et al., 1996). To date six genes, ULBP1–6, have been identified as belonging to the ULBP family. These molecules are 55–60% homologous in their amino-acid sequences, and are equally distantly related to MICs or MHC (around 20% sequence similarity Cosman et al., 2001; Chalupny et al., 2003; Eagle et al., 2009). Functionally, the ULBPs are similar to MICA/B in that they do not bind β2-microglobulin or present antigenic peptides (Groh et al., 1996), however, in contrast to MIC proteins, they lack an α3 domain and many of them attach to the membrane via a GPI-anchor.

MICA/B are highly polymorphic proteins, indeed more than 70 MICA and 30 MICB sequences have been described to date (Steven GE Marsh, Anthony Nolan Research Institute, http://hla.alleles.org/terms.html) and a number of diseases have been reported to be associated with MIC gene polymorphisms, including ankylosing spondylitis, Behçet’s disease, psoriasis, type I diabetes, and Addison’s disease (Stephens, 2001). The sequence variation that gives rise to these alleles occurs throughout the protein, but MICA polymorphism is often classified into several groups of alleles according to a microsatellite polymorphism in the TM region. Strikingly, one of these groups, known as MICA5.1, is highly frequent in multiple human populations worldwide. MICA 5.1 alleles (for example, MICA*008) contain a frame-shift mutation before the TM region that leads to an early stop codon (Ota et al., 1997). A number of single nucleotide polymorphisms in both promoter regions and coding sequences of the RAET1/ULBP genes has been described (Antoun et al., 2010), although the number of variant transcripts generated by these mutations is limited (**Figure 1**). The significance of polymorphism for receptor binding is unclear since NKG2D is known to bind to its ligands by adapting the homodimer to the α1/α2 helices of the monomeric ligand (Strong and McFarland, 2004). The interface between the receptor and its distinct ligands are stabilized by hydrophobic interactions and hydrogen bonds and, interestingly, different residues are involved in the interaction with the different ligands in both human (Li et al., 2001; McFarland et al., 2003) and murine systems (Wolan et al., 2001). This plasticity in the NKG2D/NKG2D-ligand interaction allows the receptor to recognize a large number of diverse molecules in the absence of an important conformational modification upon ligand binding (Radaev et al., 2001). In the context of MICA polymorphism, it is striking to note that even though some allelic amino-acid changes are quite dramatic (Pro/His) and some polymorphic residues lie very close to the NKG2D contact site, only one dimorphic variant of MIC has been shown to affect the affinity with which MICA binds to NKG2D and this is a conservative substitution, Met or Val at residue 129 (Steinle et al., 2001). Strikingly, while NKG2D recognizes all its ligands with sufficient affinity to signal NK cell activation, a number of viral immune evasion proteins do discriminate between members of the ULBP and MIC families, binding either MICA or MICB and some, but not all, of the ULBPs. These observations are consistent with our hypothesis that the biochemical similarities and differences among NKG2D-ligands can be functionally important and that these characteristics are independent of the genetic family to which the NKG2D-ligands pertain.

## EXPRESSION OF NKG2D-LIGANDS

Although mRNA for both MICA/B and ULBP proteins can be found in some normal cells (Cosman et al., 2001; Schrambach et al., 2007), there is general consensus that the levels of NKG2D-ligand expression at the cell surface of the vast majority of healthy cells, are either null or low and clearly below that needed to activate immune cells expressing NKG2D receptors. Instead, the expression of these molecules is upregulated when the cells suffer different types of stress, notably tumoral transformation, viral and bacterial infection, and in autoimmune diseases. A large variety of primary tumors and tumor-derived cell lines express NKG2D-ligands and in the last few years, numerous comprehensive reviews have been published on the involvement of NKG2D-ligands in cancer (Nausch and Cerwenka, 2008). Thus, only some aspects of the complexity of this topic are discussed here. The molecular mechanisms involved in the regulation of expression of these molecules upon stress are not clear, however it has been reported that heat shock, oxidative stress, DNA damage, proteasome inhibition, and histone deacetylases lead to an increased expression of NKG2D-ligands at the cell surface (for review, see Mistry and O’Callaghan, 2007; Gonzalez et al., 2008 and references therein). Of particular interest is that the effect of all those stress stimuli leads to differential expression of the various NKG2D-ligands, depending on the cellular type and/or its metabolic status. For example, proteasome inhibition specifically upregulated ULBP2 in Jurkat cells (Vales-Gomez et al., 2008), but ULBP1 in head and neck squamous cell carcinoma (HNSCC) cells (Butler et al., 2009). What seems clear is that, in the context of stress, NKG2D-ligand expression and release are regulated at many cellular levels including transcriptional, post-transcriptional [mRNA stability and micro-RNAs (mi-RNAs) Stern-Ginossar et al., 2008; Heinemann et al., 2012], and post-translational (protein modifications, trafficking, and shedding). As mentioned above, cytokines also affect the expression of NKG2D-ligands: while IFN-γ downregulates MICA and ULBP2 (Schwinn et al., 2009; Yadav et al., 2009), IFN-α upregulates its expression (Jinushi et al., 2003; Zhang et al., 2008). Another level of complexity is that expression of a particular ligand in different types of cancer can be associated with better or worse prognosis (Wu et al., 2004; Li et al., 2009; McGilvray et al., 2009,^2010^; Paschen et al., 2009; Nuckel et al., 2010). It thus seems plausible to suggest that the diversity of NKG2D-ligands detected in different tumors reflects, on one hand, the complexity in the regulation of their expression at the cellular and tissue microenvironment level and, on the other, the contribution from the biochemical properties conferred by diversity and polymorphism. Evolution of NKG2D-ligands has been most probably driven by pathogen pressure, as illustrated by the example of HCMV (see below), however, the resulting benefit ensuring good recognition of infected cells can result in differential responses against tumors, from direct killing of the transformed cell to evasion of the immune response. Recognition of NKG2D-ligands, either in soluble form or at the cell surface, can result in downmodulation and degradation of the activating receptor (Groh et al., 2001; Roda-Navarro and Reyburn, 2009) and differences have been observed whether the interacting ligand was membrane bound or soluble, the former being more potent for receptor inactivation (Salih et al., 2002; Coudert et al., 2005).

In the next sections, we will review the cell biology of NKG2D-ligands and group them according to their biochemical features, with the idea that this way of thinking may provide useful insights into the biology of these molecules. All the biochemical features of NKG2D-ligands discussed in the next sections are summarized in **Table 1**.

**Table 1:** NKG2D-ligand biochemistry and cell biology.

	ULBP1/RAET1I	ULBP2/RAET1H	ULBP3/RAET1N	MICA/long TM – cytoplasmic tail	MICA/shortTM – cytoplasmic tail	MICB
Stability at the cell surface^1^ (hour)	<4	>4	>4	>4	>4	<4
DRMs (%)	~100	~100	~100	~15	~70%	~15%
Maturation^2^	>2 h	>2h	30 min	30 min	NA	>2 h
Release						
Exosomes	Low^3^	Low^4^	Y	Low^4^	Y	Low
Soluble	Low^3^	ADAM	Low	ADAM/MMP14	Low	ADAM
Viral evasion						
HCMV-UL16 (downmodulation)	Y	Y	N	N	P	Y
HCMV-UL142 (downmodulation)	N	N	Y	Y	N	N
HIV-Nef (downmodulation)	Y	Y	NA	Y^5^	Y^5^	NA

*^y^, yes; N, no; P, partially; NA, not addressed.*

1 Time to reach 50% reduction in cell surface expression after blockade of protein synthesis and recycling.

2 50% of protein mature after the indicated time, as evaluated in pulse-chase experiments.

3 In general, present at low levels and in exosomal fractions of certain cells.

4 Enhanced by metalloprotease inhibitors.

5 The alleles were not specified in the study.

## BIOCHEMICAL FEATURES AND CELL TRAFFICKING OF NKG2D-LIGANDS

The classification of NKG2D-ligands according to their genetic location correlates with the presence of an α3 domain and with the primary mode of membrane attachment of these molecules: MICA and MICB are TM proteins whereas the most studied ULBPs (ULBP1–3) are GPI-linked molecules (**Figure 1**). However, this classification is not so straightforward since ULBP4 is a TM protein (Chalupny et al., 2003; Bacon et al., 2004) and ULBP2 and 5 have recently been shown to have the potential to be expressed as either a GPI or a TM protein (Ohashi et al., 2010; Fernandez-Messina et al., 2011) demonstrating that, depending on cellular context a single mRNA sequence can encode proteins with two types of membrane attachment.

Despite these caveats, the observation that murine NKG2D-ligands are also expressed as either TM or GPI-linked molecules has led to the suggestion that the conservation of a GPI-anchor in some, but not all, NKG2D-ligands might be functionally important. However, the nature of this putative functional significance remains unclear. In the mouse, GPI-anchored proteins have modest to low affinities for NKG2D, whereas the ligands possessing TM-cytoplasmic domains have high affinity (O’Callaghan et al., 2001), but the human NKG2D-ligands do not seem to conform to this pattern (Strong and McFarland, 2004). Another possibly relevant difference between GPI-anchored and TM proteins is that GPI-anchored proteins usually associate with detergent resistant membranes (DRMs), which are regions of the membrane enriched in sphingolipids and cholesterol. Indeed, the majority of ULBP1–3 proteins are recruited to these regions of the membrane (Fernandez-Messina et al., 2010) while only a low proportion of MICA/B appears in DRMs (Aguera-Gonzalez et al., 2009). However, this statement does not seem to be a general rule for MICs, since a high proportion of MICA*008 molecules are also recruited to DRMs (Ashiru et al., 2010). Clustering of the GPI-anchored ULBPs within lipid rafts, that are known to polarize to the site of interaction between the NK cell and the susceptible target cell (Lou et al., 2000), could increase the avidity of interaction of these molecules with the NKG2D receptor. In support of this hypothesis, Martinez et al. (2011) have observed that redistribution of ULBP1 outside of DRM, through the replacement of the GPI linkage in ULBP1 by the TM region of CD45, resulted in diminished NK cell responses to target cells expressing these molecules. In contrast, a naturally occurring TM form of ULBP2 was as capable of enhancing NK cell activation as the GPI-linked form of ULBP2 (Fernandez-Messina et al., 2011). Consistent with this observation, a mutant MICA molecule that could not be recruited to DRMs triggered NK cell lysis comparably to wild-type MICA (Aguera-Gonzalez et al., 2011). The reasons for the different results obtained in these three studies are not clear, but one obvious difference is that each paper analyzed a different NKG2D-ligand. It would be interesting to investigate the contribution of these distinct membrane anchors to the distribution and properties of specific ligands on target cells.

Another statement that does not seem to hold true when NKG2D-ligands are compared, is that possession of a GPI-anchor leads to a stable linkage to the exoplasmic leaflet of the lipid bilayer, resistant to proteases and lipases. In fact, it is known that GPI-anchored proteins can be endocytosed (for review, see Sabharanjak and Mayor, 2004; Maeda and Kinoshita, 2011). Indeed, analysis of the stability of the NKG2D-ligand at the cell surface reveals important differences in the half-life of these molecules that do not correlate with the form of membrane anchoring: MICA and ULBP2, 3 are quite stable at the plasma membrane, ULBP1 disappears with faster kinetics (Fernández-Messina, 2011) and MICB has a very short half-life at the plasma membrane (Aguera-Gonzalez et al., 2009).

## RELEASE OF NKG2D-LIGANDS

The presence of soluble NKG2D-ligands in serum from cancer patients and the persistent engagement of the NKG2D receptor has been related with an impairment of NKG2D-mediated cytolytic functions (Salih et al., 2008 and references therein). Thus, the mechanisms underlying NKG2D-ligand release have been intensively studied in the last few years and, here again, marked similarities and differences between NKG2D-ligands that do not correlate with genetic family to which the ligands belong have been observed. MICA molecules with long TM and cytosolic domains (Salih et al., 2002), MICB (Boutet et al., 2009), and ULBP2 (Waldhauer and Steinle, 2006) are shed after proteolytic cleavage mediated by metalloproteases, whereas MICA*008 (short TM and cytoplasmic tail) and ULBP3 molecules are released as full-length proteins located in exosomes (Ashiru et al., 2010; Fernandez-Messina et al., 2010). Much less ULBP1 is released than either ULBP2 or 3, when comparing supernatants from the same cellular system. These data support the existence of intrinsic differences between the biochemistry of the NKG2D-ligands that do not correlate with a MIC/ULBP classification, and revealed that the integrity of cellular trafficking can have a marked influence on the behavior of the different NKG2D-ligands. Treatment of cells with metalloprotease inhibitors, led to the recruitment of ULBP2 into exosomes (Fernandez-Messina et al., 2010). Similarly, MICA*019, normally shed as a soluble molecule, can also be found in exosomes after metalloprotease inhibition (Fernández-Messina, 2011). Thus, ULBP2 and MICA*019 could traffic to multivesicular bodies for incorporation into exosomes, but do not complete this journey because of cleavage by metalloproteases. In fact, all the ULBPs were found in exosomes derived from placental tissues (Hedlund et al., 2009) and ULBP1 in dendritic cell-derived exosomes (Viaud et al., 2009). Membrane compartmentalization of metalloproteases and NKG2D-ligands may represent one level of regulation of trafficking and shedding of the NKG2D-ligands since recruitment of both sheddase and ligand to DRMs was crucial for efficient proteolytic release of MICB (Boutet et al., 2009). Moreover, palmitoylation has been shown to regulate the recruitment of MICA to DRMs and also to markedly influence shedding (Aguera-Gonzalez et al., 2011). However, other levels of post-translational regulation of this process are likely to exist, for example the thiol-isomerase endoplasmic reticulum protein (ERp)5, has also been reported to modulate MICA shedding (Kaiser et al., 2007).

The consequences of all these different mechanisms for NKG2D-ligand release are not trivial with regard to immune recognition. It is important to remember here that although MIC and ULBP molecules can be potent activating ligands for NKG2D, persistent chronic engagement of NKG2D upon interaction with surface ligands leads to receptor downmodulation and loss of function (Coudert et al., 2005; Oppenheim et al., 2005; Wiemann et al., 2005). Thus, it is difficult to predict how the expression of NKG2D-ligands could affect the intensity of an immune response of a cancer patient since this will depend on multiple factors including the presence of NKG2D-ligands in serum and, as discussed above, the particular cytokine milieu that could oppose or enhance NKG2D downmodulation by serum NKG2D-ligands. Further, the biochemical forms of NKG2D-ligands found in patients’ sera may vary firstly, depending on the particular alleles encoded in that individual’s genome and, secondly, on the effect that tumor transformation could have on the cellular pathways needed for the molecule to reach either the cell surface or the extracellular milieu. So, while the MICA*008 allele and ULBP3 are generally released in exosomes, many MICA/B molecules and ULBP2 can be shed as either soluble proteins or in exosomes depending on the metalloprotease activity in the cell (see above). This distinction is important since TM proteins at the surface of exosomes are presented in the same orientation as those at the cell surface (Simons and Raposo, 2009). In this sense, an exosome can be considered as a nanoparticle presenting multimeric NKG2D-ligands and it has been reported that exosomal NKG2D-ligands are more potent downmodulators of the NKG2D receptor than cleaved, soluble molecules (Ashiru et al., 2010; Fernandez-Messina et al., 2010). It is important to note that although MICA*008 can be released as a full-length protein in exosomes, this does not imply that it cannot be detected in patients sera since the method for detection does not distinguish between these two biochemical forms. Indeed, soluble MICA molecules can be detected in patients’ sera regardless of presumed allelic MICA differences, and in particular high sMICA levels have been found in MICA 5.1^+^ individuals (i.e., mostly MICA*008; Hue et al., 2004). Thus, the expression of NKG2D-ligands could modulate in many different manners the intensity of the immune response of a cancer patient. Each one of these possibilities has different outcomes for the immune system and, identification of the particular route used to release NKG2D-ligands could provide information on immune system integrity and/or cellular routes affected in the tumor.

## NKG2D-LIGANDS, CELL BIOLOGY, AND PATHOGEN IMMUNE EVASION STRATEGIES

The release of NKG2D-ligands from cells discussed above is thought to be a major mechanism for tumor cell evasion of NKG2D-mediated immune surveillance. However, apart from cancer, the NKG2D system plays a role in several other pathological situations that involve some degree of cellular stress, including transplantation (Collins, 2004; Suarez-Alvarez et al., 2006), autoimmune diseases (Van Belle and von Herrath, 2009), and pathogen infection (Borchers et al., 2006). The observation that a number of pathogens, especially viruses, have developed strategies to evade NKG2D-mediated recognition shows the importance of this system to control infection. Immunoevasin discrimination between NKG2D-ligands presumably reflects the existence of important functional differences between the NKG2D-ligands. For example, the HCMV glycoprotein UL16 blocks surface expression of ULBP1, 2, 6, and MICB, but not MICA, ULBP3, or ULBP4/RAET1E (Chalupny et al., 2003; Dunn et al., 2003; Rolle et al., 2003; Vales-Gomez et al., 2003; Welte et al., 2003). However, the HCMV-UL142 protein downmodulates MICA and ULBP3, but does not affect the alleles of MICA with a short TM/cytoplasmic tail or ULBP2 (Chalupny et al., 2006; Ashiru et al., 2009; Bennett et al., 2010). Consideration of several aspects of NKG2D-ligand cell biology (**Table 1**) can provide a novel perspective that may prove useful in understanding the interaction of immunoevasins with specific NKG2D-ligands as well as providing insights into the mechanisms of action of these molecules. For example, the rate of maturation of NKG2D-ligands varies among the individual molecules, ranging from 30 min to more than 2 h for ER exit, implying that each NKG2D-ligand follows different cellular routes and/or suffers different post-translational modifications. Interestingly, in some cases this biological variability can be correlated with the interaction of NKG2D-ligands with viral immunoevasins: for example, HCMV-UL142 preferentially binds those proteins that leave the ER rapidly, while HCMV-UL16 efficiently sequesters those that share the property of slow exit from the ER. It is striking to note that UL16 also leaves the ER only very slowly (Vales-Gomez et al., 2006), paralleling the behavior of the NKG2D-ligands with which it interacts, perhaps implying that this shared trafficking property is important for the intracellular accumulation of NKG2D-ligands mediated by UL16. The selective binding of UL16 to specific NKG2D-ligands may also be influenced by the strength of the interaction between the luminal domains of the viral protein and the NKG2D-ligand (Muller et al., 2010) and it seems reasonable to propose that viruses specifically target ligands according to features such as binding and/or trafficking. A number of other viruses also target specific NKG2D-ligands including human herpesvirus-7 (HHV-7; Schneider and Hudson, 2011) that redirects ULBP1 molecules to lysosomes, and the Kaposi’s sarcoma-associated herpesvirus K5 protein that promotes degradation of MICA molecules upon ubiquitylation of a lysine motif in the cytoplasmic tail (Thomas et al., 2008). Interestingly, both the GPI-anchored NKG2D-ligand proteins and the short-tailed alleles of MICA, lacking cytoplasmic tail, are resistant to degradation by this latter mechanism. The HHV-7 U21 gene also downmodulates MICA and MICB at a post-translational step, although the mechanism underlying this phenomenon is not completely understood. In contrast, those immune evasion proteins that downmodulate multiple NKG2D-ligands, for example, HIV-Nef that acts on all the NKG2D-ligands tested (ULBP1, 2, and MICA; Cerboni et al., 2007), seem likely to target some feature highly conserved between the different NKG2D-ligands. Thus, the existence of viral immunoevasins that selectively target some, but not all, NKG2D-ligands clearly demonstrates the existence of important differences in the biochemistry and cell biology of these molecules. Moreover identification of functionally important features that correlate with susceptibility to recognition by an immunoevasin may be a useful approach to shed light on its mechanism of action.

An additional level of viral regulation of ligand expression is represented by mi-RNAs: diverse herpes and polyomaviruses encode mi-RNAs that target NKG2D-ligand mRNA, to reduce their expression (Nachmani et al., 2009). However, here again, it is possible to argue that biochemical differences between the different NKG2D-ligands may have influenced the evolution of the specificity of these mi-RNAs. For example the HCMV mi-RNA UL112 acts to reduce expression of MICB, but not MICA. It is tempting to speculate that it is advantageous for the virus to have evolved a mi-RNA able to specifically block MICB transcription because the short half-life of MICB at the cell surface would mean a rapid loss of surface protein after blockade of mRNA transcription (Aguera-Gonzalez et al., 2009). In contrast, the action of a viral protein would be required to actively sequester the much more stably expressed MICA protein.

## CONCLUDING REMARKS

Review of the biochemical properties of the different NKG2D-ligands supports the idea that evolution of these molecules was significantly driven as a result of selective pressure exerted by a range of stress signals and pathogen infections: if an insult resulted in blockade of a particular cellular pathway, obstructing the expression of an NKG2D-ligand, the existence of a different ligand that could follow an alternative route to make the cell visible to the immune system would be an advantage. If, however, the actual set of ligands for the NKG2D receptor was the result of a process of evolution for diversity, it is not surprising that the biochemical properties of a given NKG2D-ligand do not necessarily reflect the behavior of other members of its genetic family. This kind of consideration reinforces the necessity for further studies to analyze the cell biology and biochemistry of the individual NKG2D-ligands in detail. This information will be especially important for understanding the role of this system in disease, in particular the detection of specific soluble NKG2D-ligands in the sera of patients suffering cancer. Although in many cases correlations between the levels of soluble ligands and disease progression have been reported, it is reasonable to suggest that identifying the NKG2D-ligand genotype of the patient, in particular long versus short MICA molecules, can contribute to their use as biomarkers. In this sense, it might well be worthwhile to revisit previous reports looking at which particular NKG2D-ligand was studied, not whether it was a MIC or a ULBP. Given that aberrant expression of NKG2D-ligands is involved in the pathogenesis of both cancer and various autoimmune diseases, learning the lessons of NKG2D-ligand cell biology may represent a useful approach to develop novel strategies to permit selective or general manipulation of this system in disease.

## Conflict of Interest Statement

The authors declare that the research was conducted in the absence of any commercial or financial relationships that could be construed as a potential conflict of interest.
